# Imaging complex ventral hernias, their surgical repair, and their complications

**DOI:** 10.1007/s00330-018-5328-z

**Published:** 2018-03-12

**Authors:** Steve Halligan, Sam G. Parker, Andrew A. Plumb, Alastair C. J. Windsor

**Affiliations:** 1 Centre for Medical Imaging, University College London, Charles Bell House, 43-45 Floey Street, London, W1W TS UK; 2 The Abdominal Wall Unit, Department of Surgery, University College Hospital, Euston Road, London, NW1 2BU UK

**Keywords:** Hernia, Ventral, Abdominal Wall, Hernia, Abdominal, Incisional Hernia, Tomography, Spiral Computed

## Abstract

**Abstract:**

Complex ventral hernia (CVH) describes large, anterior, ventral hernias. The incidence of CVH is rising rapidly due to increasing laparotomy rates in ever older, obese and co-morbid patients. Surgeons with a specific interest in CVH repair are now frequently referring these patients for imaging, normally computed tomography scanning. This review describes what information is required from preoperative imaging and the surgical options and techniques used for CVH repair, so that radiologists understand the postoperative appearances specific to CVH and are aware of the common complications following surgery.

**Key Points:**

*• Complex ventral hernia (CVH) describes large abdominal wall hernias (e.g. width ≥10cm).*

*• CVH patients are being referred increasingly for preoperative and postoperative imaging.*

*• Imaging is pivotal to characterise preoperative morphology and quantify loss of domain.*

*• Postoperative imaging appearances are contingent on the surgical methods used for CVH repair.*

*• Postoperative complications are depicted easily by imaging.*

## Introduction

Examination of a simple inguinal hernia was often our first introduction to surgical practice and their repair is usually straightforward. However, the current surge in complex ventral hernia (CVH) is changing this belief. CVH describes large, anterior, incisional hernias (alternatively known as “giant” ventral hernias). Ventral hernia follows 20% of laparotomies, resulting in a 5% lifetime risk [1]. Incidence is growing rapidly due to rising laparotomy rates in increasingly older, obese and co-morbid patients. While bariatric weight-loss procedures hog the limelight, other consequences of obesity, such as CVH, receive much less publicity. A 2013 article estimated 348,000 ventral hernia repairs occurred annually in the USA at a cost of $3.2 billion [2]. Successful repair of large hernias demands specific expertise, and specialists in abdominal wall reconstruction are emerging. These surgeons are asking radiologists to image CVH patients but a recent systematic review by the authors found very little available data describing radiology of CVH [3]. To rectify this, our review describes preoperative and postoperative imaging of CVH and their complications.

### The clinical problem

While most ventral hernias are repaired easily, CVH poses specific problems. The anatomical defect is large and complex, and surgeons frequently encounter morbid obesity, infection, enterocutaneous fistula and stomas. Accordingly, recurrence is common, reaching 27% at just 1 year for patients whose BMI exceeds 35, versus 8.3% for those under 25 [4]. A wide variety of surgical techniques are available, along with a sizeable range of mesh, used to close the defect. Lack of consensus regarding the optimal surgical approach and material complicates the issue further.

Why should we repair CVH if surgery is so risky? CVH causes chronic back pain, abdominal discomfort and poor respiratory function. Patients with large, heavy hernias are unstable and often wheelchair bound [5]. CVH inevitably means poor quality of life [6]. Furthermore, hernia research is neglected: Professor Michael Rosen, Director of the Cleveland Clinic Hernia Centre, has said, “When hernia surgery goes wrong, it results in some of the most despondent, challenged patients with the worst quality of life who are desperate for improvements. This is hard to imagine, because hernia surgery is probably the most common surgery performed by general surgeons”. Going on to state, “Hernia disease has been one of the most neglected procedures in the field of general surgery. There has been very little innovation during the past 50 years” [7]. Prof. Poulose of Vanderbilt University Medical Centre has stated, “If a patient has colon cancer he can expect virtually the same treatment anywhere in the world but if a patient has an abdominal wall hernia, his treatment can vary significantly between countries, states, hospitals and even within the same practice” [8].

Surprisingly, there is no generally accepted definition of CVH [9]. Multiple different and overlapping classification systems have been identified [10]. The European Hernia Society classifies CVH by location, defect size, reducibility, symptoms, and recurrence [9] and an expert meeting reached consensus on 22 individual patient and hernia variables that could be used to define CVH [10]. Slater and co-workers [10] stated that CVH could be defined as a “large-sized abdominal wall hernia”, defining this as a transverse defect with a diameter of 10 cm or more, while the “abdominal” location could include midline ventral, parastomal, lateral and lumbar locations. In terms of size, they defined CVH as “loss of domain” of 20% or more. Other authors have used a figure of 30% [11]. Loss of domain is increasingly important and describes the ratio of the hernia sac volume to the residual abdominopelvic cavity [12]. A ratio of 20% or more means that one-sixth or more of the abdominopelvic content is within the hernia sac rather than the abdominopelvic cavity. Large hernias decrease abdominal wall elasticity, and cause muscular atrophy and diaphragmatic descent. Forceful return of viscera to the abdominopelvic cavity can precipitate cardiorespiratory impairment or abdominal compartment syndrome [1].

### Surgical techniques

Two innovations have exerted profound influence on CVH repair: the development of prosthetic mesh able to cover large defects and “component separation”, popularised by Ramirez and co-workers [13]. Both are usually combined for large CVH.

### Mesh type and position

A prosthetic is required for large defects. Numerous materials are available but divide broadly into synthetics (e.g. polyester, polypropylene, polytetrafluoroethylene) biologicals and biosynthetics. While biologics require processing to remove cells, biosynthetics are tissue scaffolds made from polymerisation of biochemical molecules. Prosthetics are usually deployed as a “mesh” whose pores allow tissue ingrowth, ultimately causing incorporation (vs encapsulation). Incorporation supports the abdominal wall and reduces recurrence [14]. Biologicals are tissue derived collagen and extracellular matrix (human, porcine, bovine). Their “natural” origin is claimed as a physiological advantage and they probably perform better in contaminated fields. Unit costs for biologicals are significant but they may prove cost-effective ultimately [2].

**Fig. 1:**
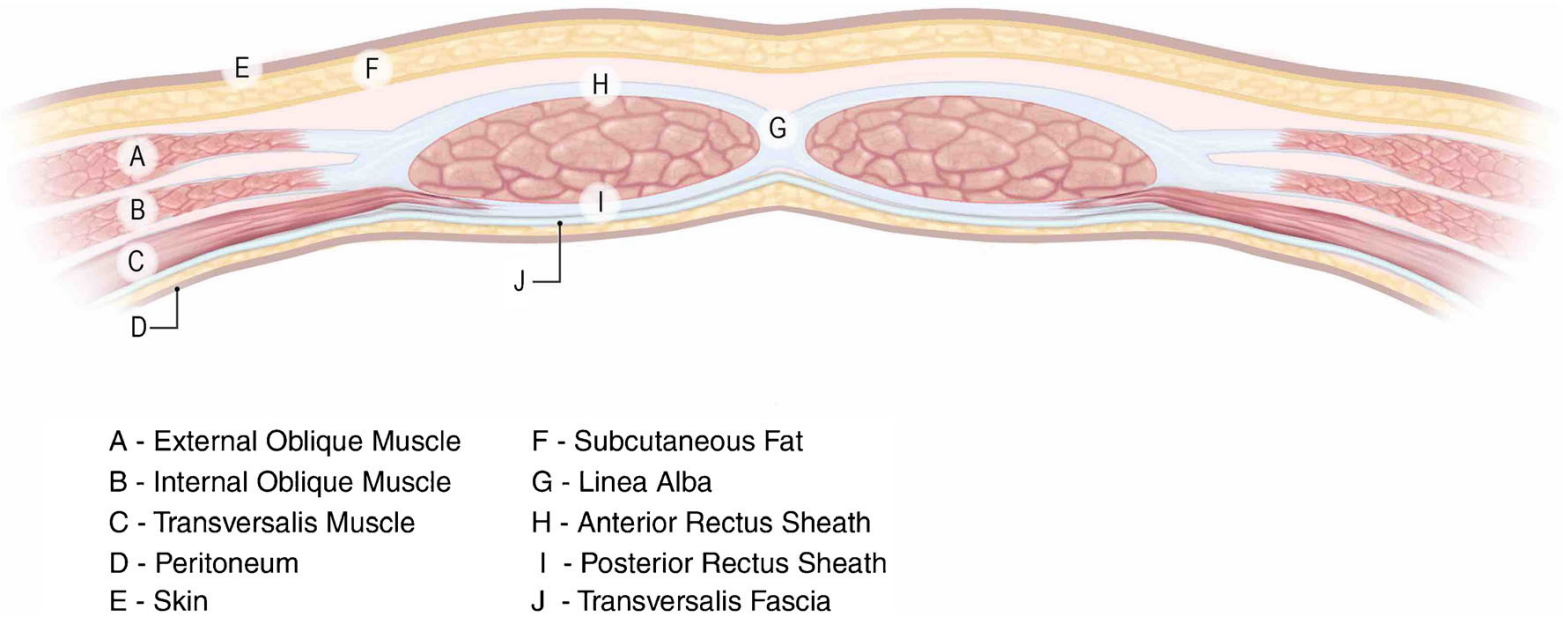
Diagrammatic representation of the axial anatomy of the anterior abdominal wall above the umbilicus, with appropriate labels

**Fig. 2:**
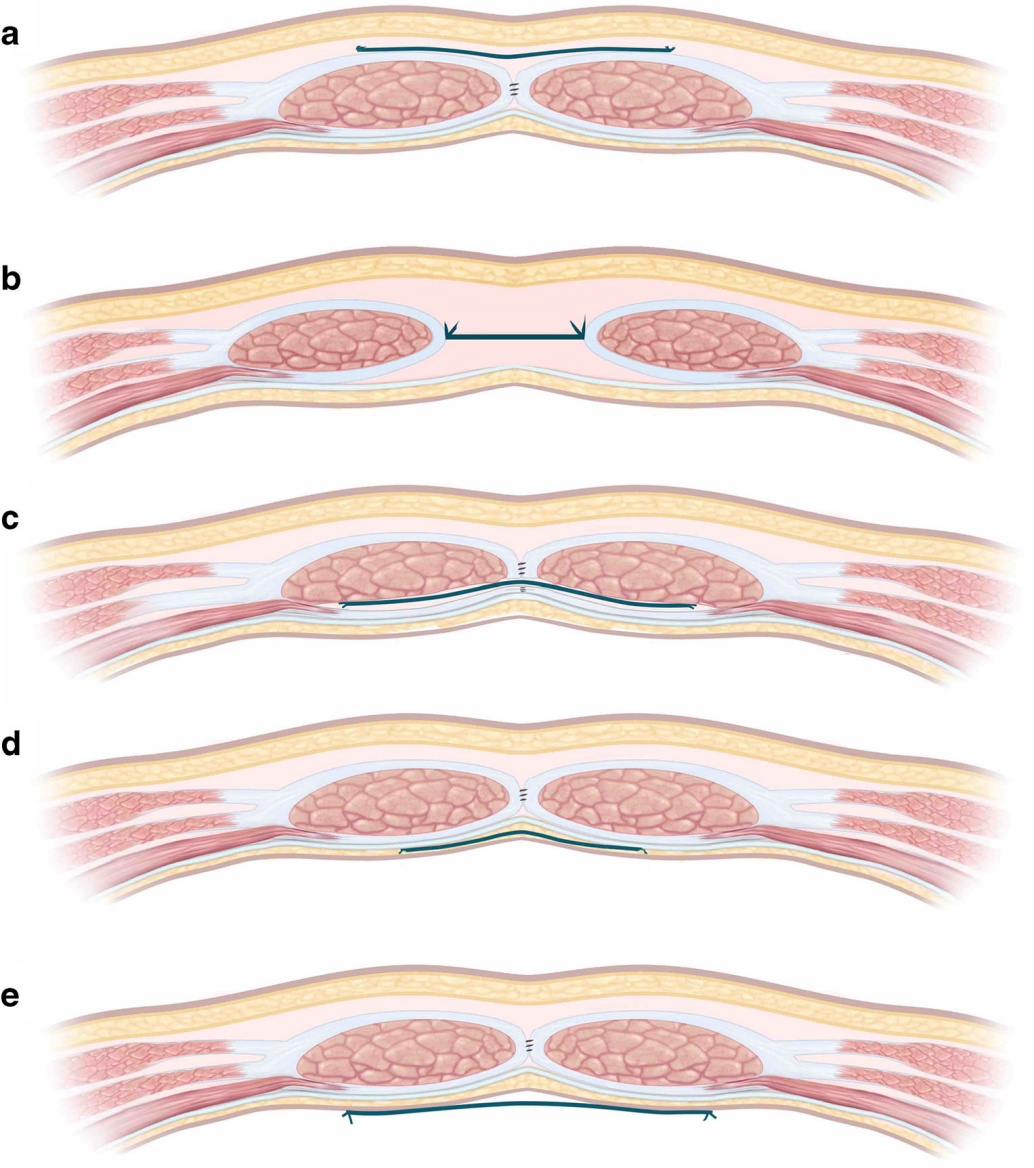
Diagrammatic representation of the planes within which prosthetic mesh may be deployed and the correct terminology associated with each placement. **a** onlay, **b** inlay, **c** sublay, **d** underlay, **e** intraperitoneal

It is important that radiologists understand abdominal wall anatomy since mesh may be deployed in numerous anatomical planes (Fig. 1), which influences imaging appearances. Surgical nomenclature is confused currently [15]. Figure 2 shows the terminology used by the authors [16, 17]. “Onlay” (alternatives “overlay”, “subcutaneous”) refers to mesh placed *anterior* to the rectus sheath and/or the external oblique muscle. “Inlay” (alternative “interposition”) refers to mesh placed *between* the separated rectus muscles. Inlay meshes “bridge” the fascial defect (whereas other positions overlap the defect), are cut to the same size and sutured to its circumference. Inlay meshes are technically easy and relatively tension free but often become detached. Patients may also believe their hernia has not been treated adequately because there is no muscular covering. Inlay is now superseded by more advanced procedures. Presently, optimal treatment of CVH is rectus approximation combined with an overlapping mesh. However, inlay may be the only option for very large defects.

A “sublay” repair (alternative “retro-rectus”) describes mesh placed immediately *posterior* to the rectus muscle, but anterior to its posterior fascia, whereas “underlay” (alternative “pre-peritoneal”) is posterior to the muscular fascia but anterior to the peritoneum. Finally, “intra-peritoneal” (alternative “intra-abdominal”) describes mesh *posterior* to the peritoneum, within the abdominopelvic cavity.

Plane choice depends largely on experience. Onlay, inlay and intra-peritoneal placement is technically easy, especially for small hernias, whereas sublay and underlay placement necessitate extensive dissection, especially if component separation and transversus abdominis release are required (*see below*).

**Fig. 3:**
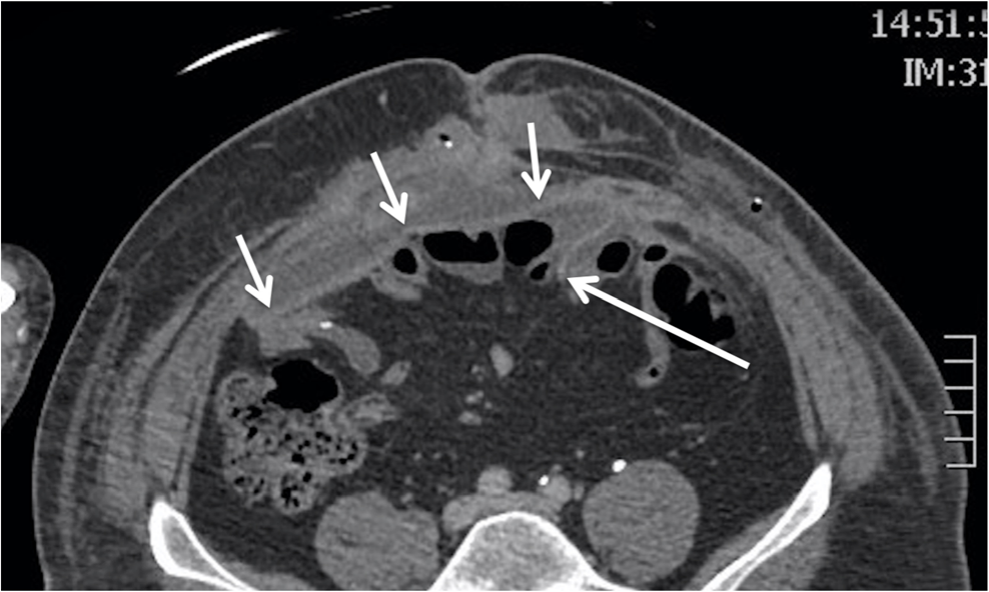
Postoperative axial CT showing an intraperitoneal mesh (*short white arrows*). The mesh has become detached (*white long arrow*), with the left lateral edge coming to lie several centimetres deep to the anterior abdominal wall. This offers the opportunity for recurrence at this site and may also cause deep adhesions

Not only does mesh position affect the radiological appearances but it also influences the development of mesh-related complications and mechanism of any hernia recurrence [18]. For example, intra-abdominal or inlay meshes may become detached laterally, especially if tacked inadequately (Fig. 3). Detachment does not happen with onlay or sublay repairs because mesh is encapsulated within a plane rather than being tacked to its margin. Abdominal viscera are in direct contact with intraperitoneal mesh, which encourages small bowel perforation, fistula and adhesions. To counter this, some meshes are coated posteriorly but uncoated anteriorly, to encourage ingrowth. Alternatively, omentum is interposed between mesh and viscera. Conversely, onlay mesh is more associated with superficial wound infection [19].

### Component separation

**Fig. 4:**
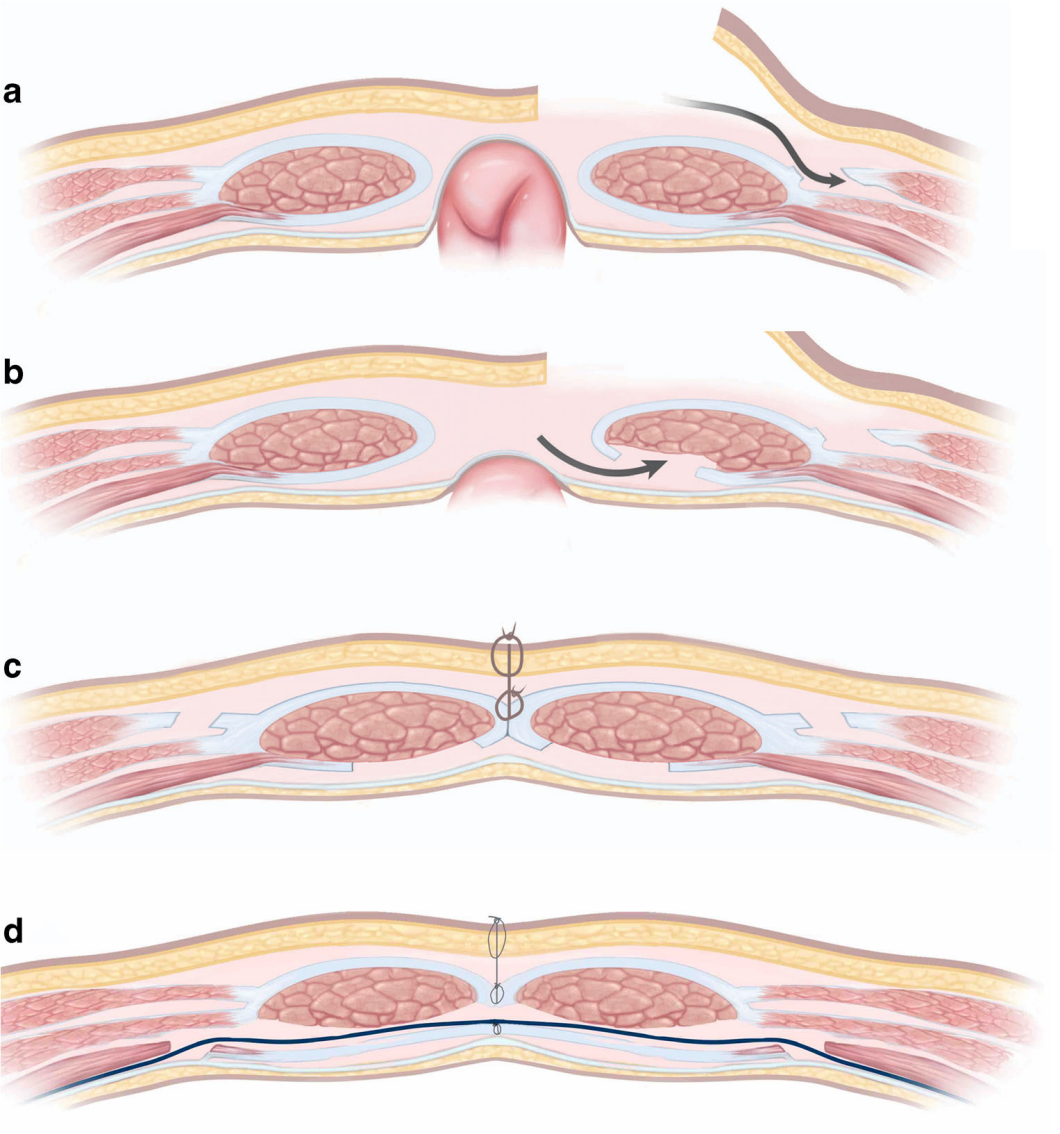
Diagrammatic representation of component separation. **a** “Anterior” component separation. A longitudinal incision is made into the external oblique aponeurosis, just lateral to the rectus sheath. The external and internal obliques are then separated from each other, allowing a medial slide of around 10 cm. **b** Further closure can be achieved by separating the posterior rectus sheath from the muscle and/or transecting the transversus. **c** The finished procedure. In many cases a mesh would also be used to strengthen the repair. **d** “Posterior” component separation achieved by longitudinal incision along the transversus muscles, the “Transversus abdominus release”. This repair has been strengthened by a mesh in the “sublay” position

Most CVH cannot be closed simply by approximating the medial rectus muscles, because the defect is too large and tension would be excessive. Ramirez and co-workers [13] popularised “component separation”, whereby abdominal wall muscles are dissected in order to facilitate their mobility. Longitudinal incision into the aponeurosis of the external oblique and dissection from the adjacent internal oblique allows the muscles to slide medially, achieving coverage with less tension (Fig. 4). Additional longitudinal incision along the medial edge of the posterior rectus sheath allows separation of this muscle from the posterior rectus sheath, achieving extra medial advancement. A more recent variant of such “posterior” component separation involves releasing the transversus abdominis from its attachment to the posterior rectus sheath (Fig. 4). This avoids the need for large skin flaps, minimising devascularisation of overlying skin and achieving less wound morbidity.

## Preoperative imaging

Preoperative imaging aims to define CVH morphology, content, abdominal muscular quality and identify any complication(s) that would compromise repair (since a large proportion will be recurrences). Table 1 describes the items that the authors include routinely in their reports. Imaging these patients is challenging because morbid obesity is the rule rather than the exception. For this reason, CT assumes prominence; the authors too frequently find patients exceed magnetic resonance imaging (MRI) bores and/or their defect too extensive for effective sonography. Intravenous contrast is probably unnecessary in most cases.

**Table 1:** Suggested dataset for the preoperative radiological reporting of complex ventral hernia

Item reported	Description	Reason for reporting
Anatomical location	Hernia through the linea alba or not? If not, the hernia is described as “lateral”. If midline, give approximate distance from the xiphisternum and symphysis pubis.	Surgeons will usually know the precise anatomical hernia site via clinical examination but occasionally radiology will reveal unexpected information.
Content	Viscera within the hernia and whether this appears normal, e.g. is bowel incarcerated/ischaemic?	To forewarn the surgeon which viscera will be encountered and whether they are diseased or not.
Defect dimensions	Maximum width × length (cm). Some workers also report hernia area (cm^2^) but the authors’ surgeons do not find this useful.	Needed to estimate the required mesh size. Approximately 5 cm added to each figure so as to obtain adequate overlap where necessary.
Loss of domain	Hernia sac volume divided by abdominal cavity volume, i.e. hernia volume relative to the residual abdominopelvic cavity.	Provides prognostic information regarding the difficulty of reducing the hernia, abdominal closure, and the systemic compromise that might arise subsequently.
Subjective impression of muscle and quality. Anterior abdominal wall thickness	Are any muscle groups missing due to prior surgery, either completely or partially? Does the residual muscle appear thin or atrophic?	Provides up-front information regarding which muscular groups are potentially available for component separation.
Evidence of previous hernia surgery?	Presence/size/insertion plane of any previous mesh. Evidence of previous component separation and the planes involved.	Prior mesh will need explantation and prior component separation will influence the choice of planes for re-do surgery.
Muscular scarring/fascial adhesions	Are planes between abdominal wall muscles preserved? Which planes are not clear?	Prior surgery may cause scarring/adhesions between fascial layers that complicate subsequent component. separation.
Collections related to any prior mesh	Location and size, width × length × depth (cm).	Collections may indicate mesh infection, which will compromise re-do if not treated aggressively.
Abdominopelvic collections	Location and size, width × length × depth (cm).	Abdominal collections are common in these patients and risk mesh infection.
Evidence of bowel obstruction/adhesions	Loops involved and diameter.	Bowel adhesions due to prior surgery will complicate re-do surgery and risk fistulae.
A standard report of abdominopelvic viscera	As per usual reporting practice.	To identify co-existent or unexpected abdominal pathology that may compromise repair.

**Fig. 5:**
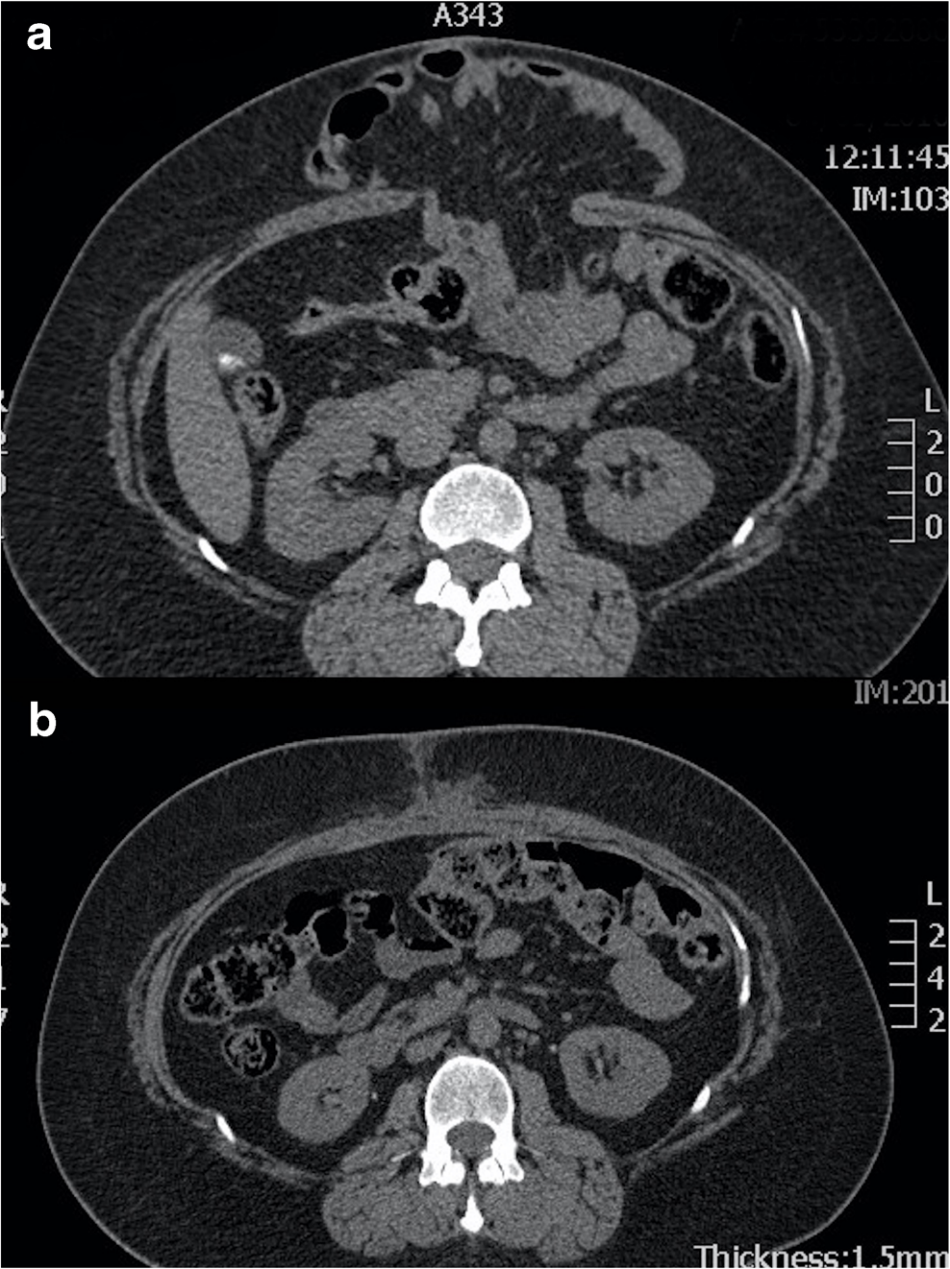
**a** Preoperative axial CT showing a ventral hernia containing small bowel. **b** Postoperative axial CT showing ventral hernia repair achieved by apposition of the rectus muscles in the midline combined with an intraperitoneal mesh

**Fig. 6:**
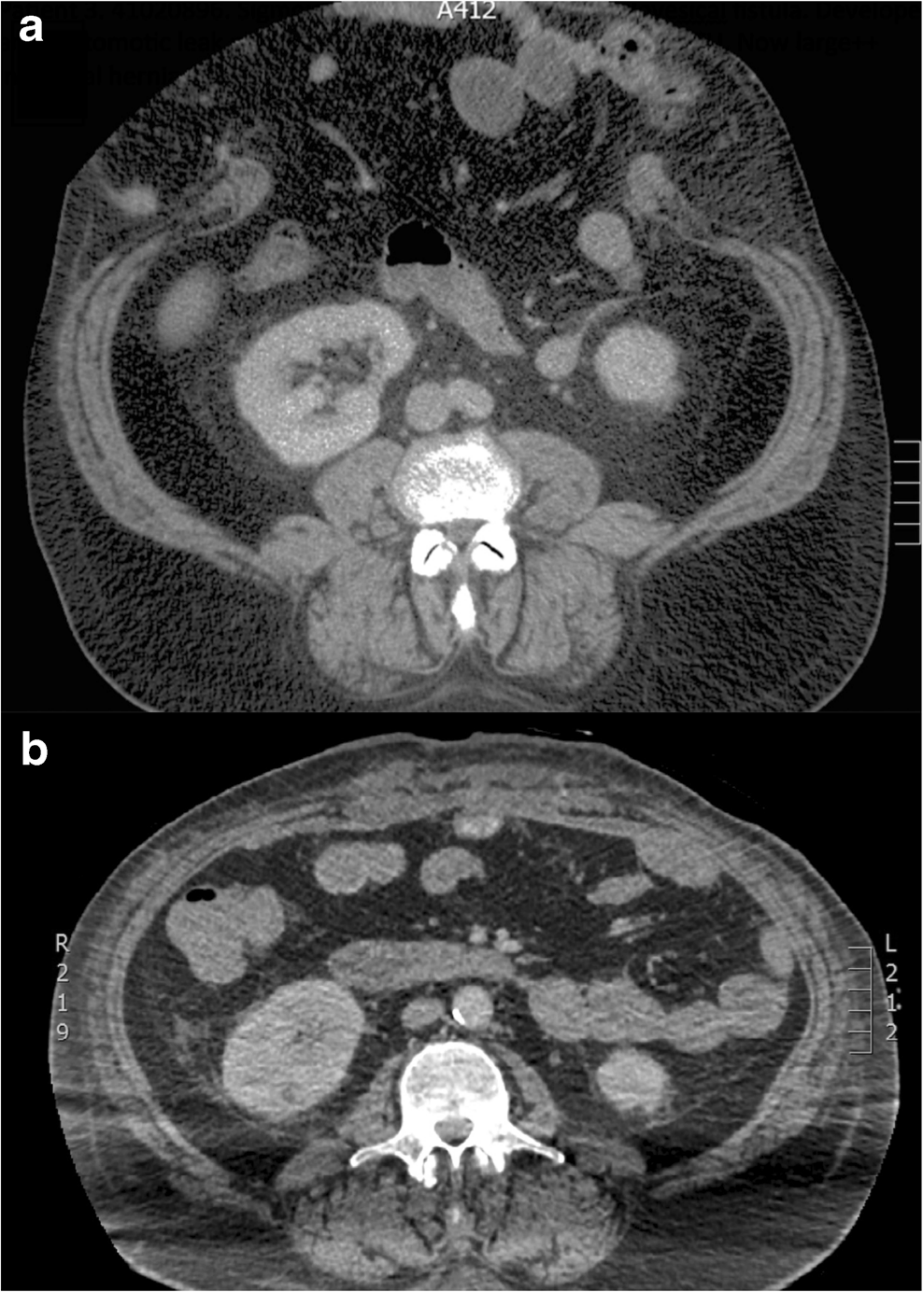
**a** Preoperative axial CT showing a huge ventral hernia following sigmoid colectomy. **b** Postoperative axial CT showing ventral hernia repair achieved by bilateral anterior component separation combined with an intraperitoneal mesh

Preoperative CT will indicate the precise location of the hernia and provides prognostic information regarding the scale of subsequent surgery [20]; for example, smaller hernias may be closed using mesh alone (Fig. 5) whereas larger defects will need additional component separation (Fig. 6). Clinical examination by the surgeon will usually provide extensive information regarding precise anatomical site but imaging may provide unexpected information. Hernia extent on CT will also help indicate the scale of subsequent abdominal and cardiovascular insult (fluid loss is considerable during extensive CVH repair), so that appropriate preoperative conditioning, intraoperative support and postoperative care can be scheduled. Ultimately, by imaging the true extent of the hernia, CT is able to indicate which can be dealt with by “general” surgeons and which need attention from a CVH specialist.

Patients seen at specialist centres have often had surgery previously and preoperative imaging can help identify the nature of this. For example, is a mesh in place and whether component separation is bilateral, the plane involved, and whether there are adhesions and/or fistula? In the authors’ experience, it can sometimes be exceptionally difficult to identify whether a mesh is in place and/or the exact plane used, and similar comments apply to which muscles have been operated upon previously. For example. The peritoneum and individual fascial layers are extremely thin on CT so the precise plane of mesh placement is often uncertain. Nevertheless, this type of information is especially important because muscle planes that have been separated previously may not be available for a re-do operation due to dense adhesions between fascial bundles (Table 1). If tacks or staples have been used to fix the mesh periphery (vs sutures) then these are easy to identify on CT as tacks are markedly hyper-attenuating and will identify the mesh margins.

The authors also provide details of which muscle groups are partially or wholly absent and a subjective impression of the “quality” of residual muscle—for example, whether it is thinned and/or atrophic. Anterior abdominal wall thickness is a metric that has been investigated but measured in multiple different ways. The most quoted work is from Blair and co-workers [21], who found that increased abdominal wall thickness was associated with postoperative success. Measurement was of the shortest distance on CT scan between the anterior rectus abdominis fascia and the skin (normally measured roughly half way between the linea alba and the semi lunar ligament), measured at umbilical level.

**Fig. 7:**
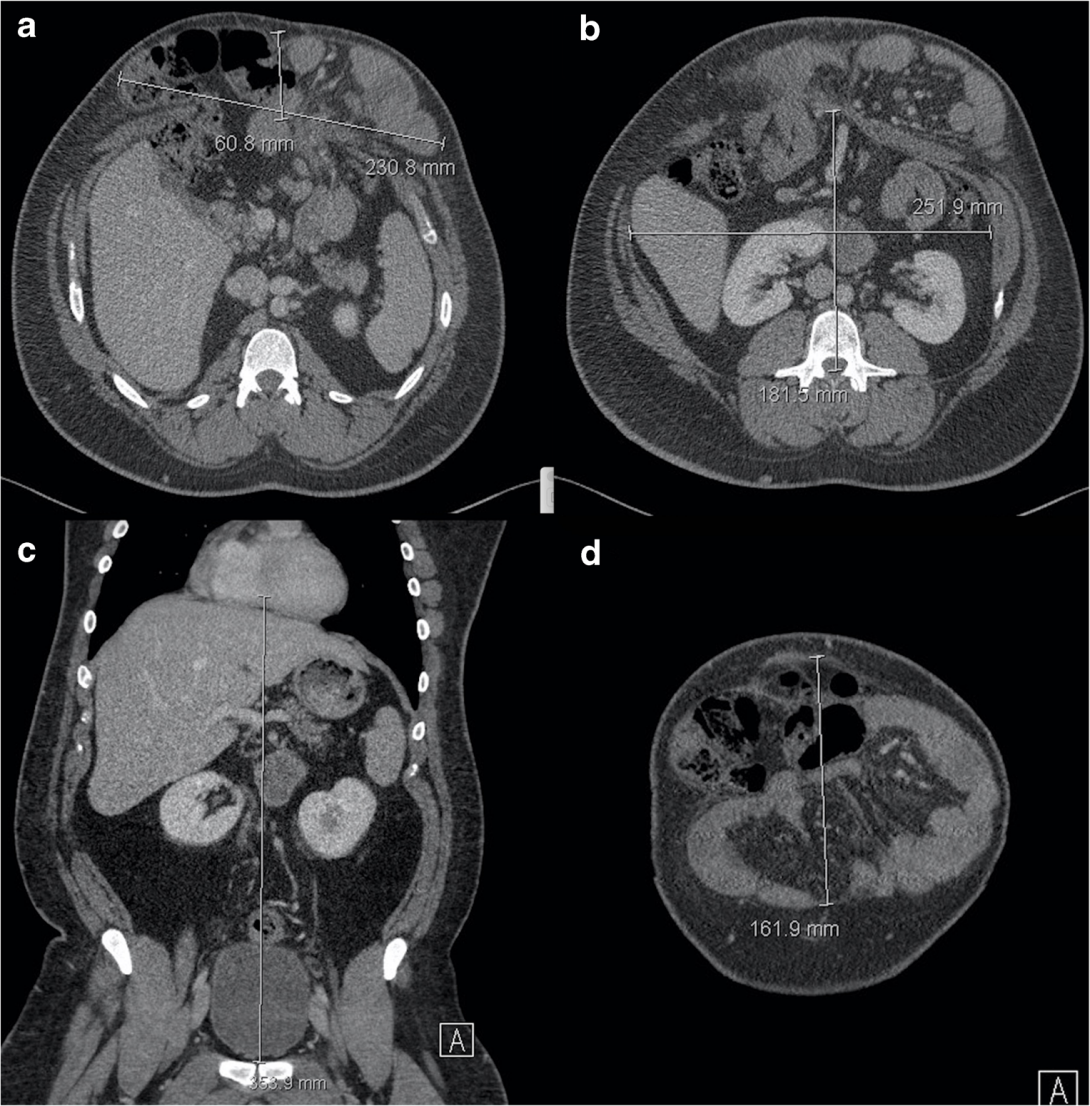
Loss of domain calculation by CT. Figures **a** and **b** show measurement of hernia (231 x 61 mm) and abdominal cavity (252 x 182 mm) width and depth respectively. Figures **c** and **d** show measurement of abdominopelvic cavity (354 mm) and hernia (162 mm) cranio-caudal length respectively. Estimated hernia sac volume (HSV) = 231 × 61 × 162 × 0.52 = 1,187,026 mm^3^. Abdominal cavity volume (ACV) = 252 × 182 × 354 × 0.52 = 8,442,645 mm^3^. Total peritoneal volume (TPV) is 9,629,671 (i.e. HSV + ACV). Loss of domain by HSV / ACV ratio is therefore = 0.14 and 12% by HSV/TPV, suggesting that hernia repair will not result in serious cardiorespiratory compromise

The CT dimension most used by surgeons is simply the maximum transverse diameter of the fascial defect, the “width”. In addition, we report the cranio-caudal dimension so that the surgeon has some advance information regarding the size of mesh that needs to be available (accounting for overlap if the mesh is not an inlay). Some workers report the cross-sectional area of the defect, but our surgeons do not find this adds anything substantial. CVH specialists will wish to know the relationship between hernia sac volume and the residual abdominopelvic cavity volume, a metric termed “loss of domain”. Loss of domain describes the extent to which the abdominal cavity has lost volume to the hernia and appears important when predicting the degree of systemic compromise that will arise when the hernia contents are returned to the abdominal cavity. Loss of domain was first calculated as the ratio arising when the hernia volume is divided by the residual abdominopelvic cavity volume, both calculated from CT scanning: 0.25 was used as the threshold to deploy preoperative abdominal tissue expanders in order to prepare for subsequent closure [12]. Subsequent workers have used a different metric, namely hernia volume divided by the total peritoneal volume (i.e. hernia volume *and* abdominopelvic volume), suggesting that >20% predicts difficulty with subsequent closure [22]. Calculating loss of domain by either method is achieved simply by measuring hernia dimensions, applying a factor to estimate ellipsoid volume (e.g. 0.52), and then doing the same for the abdominopelvic cavity (Fig. 7). Automatic segmentations to perform similar tasks have been described but are not available widely [23, 24]. Loss of domain has not been validated extensively nor are simple methods able to cope with unpredictable hernia shape. The effect of patient position on CVH morphology has not been studied, although our anecdotal experience would suggest many patients cannot lie prone.

Franklin and co-workers [20] reviewed retrospectively the preoperative CT scans of patients who had undergone component separation; they found that defect width, area and the proportion of abdominal wall circumference involved differed significantly between patients whose fascial defects were closed by re-approximation and those that could only be bridged. They suggested that preoperative CT could predict whether re-approximation was possible and therefore the likely surgical approach needed to treat CVH. Blair and co-workers [21] also reviewed preoperative CT retrospectively and found that the need for component separation, panniculectomy and incidence of postoperative complications increased with defect length, width and area. Agnew and co-workers [24] used CT to measure abdominal cavity volume preoperatively and postoperatively, and correlated this with pulmonary function, concluding that the increase in volume made possible by component separation meant that pulmonary function was not impaired. Preoperative assessment of muscular quality by imaging is probably underutilised and justifies considerable future research attention.

Some centres perform CT angiography to map perforators when considering periumbilical perforator surgery, a CT technique currently most employed for breast reconstruction using DIEP flaps [25].

## Postoperative imaging

Few articles have investigated the effect of successful CVH repair on the appearance of abdominal wall musculature. Hicks and co-workers [26] examined CT studies retrospectively, finding that the external oblique appeared to atrophy after mobilisation but that the rectus, internal oblique and transversus appeared to hypertrophy. In daily practice, postoperative imaging will usually be requested to investigate potential complications that, as noted already, are inevitable due to the high prevalence of comorbidity in these patients.

### Complications

**Fig. 8:**
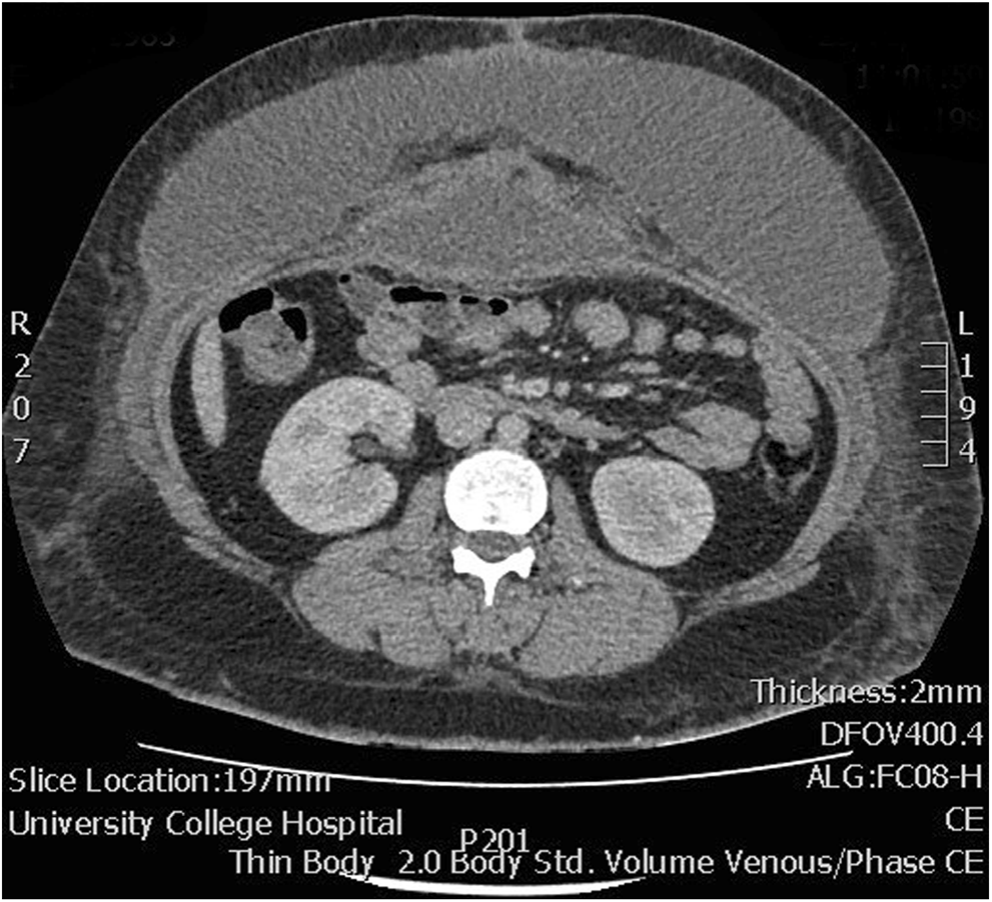
Axial CT showing a very large seroma that occurred following anterior component separation and mesh implantation

Early complications are broadly grouped into local [21] (wound dehiscence, seroma, skin necrosis, wound infection, hernia recurrence) and systemic [27] (myocardial infarction, heart failure, pneumonia, DVT, PE). Complications can also be grouped into those that are relatively specific to CVH repair and those that are common to abdominal surgery in general; the latter have been well-described previously by other authors—for example, bowel obstruction, iatrogenic perforation and fistula formation [28]. Large and extensive seromas are especially common following CVH repair due to the wide-ranging dissection needed to create abdominal flaps and pockets capacious enough to accommodate the mesh with sufficient overlap (Fig. 8). Such seromas have been reported in up to 10% of patients [29]. Seromas should be treated seriously since their infection will often compromise the repair. Chronic seromas may develop a fibrous capsule, sometimes visible on imaging, which will need surgical excision to prevent re-accumulation. Postoperative seromas may have to be distinguished from haematomas, which are also common following CVH repair, but which are identified on CT scanning by their higher attenuation and more heterogeneous content [30].

Component separation necessitates extensive dissection and undermining in order to separate muscular planes and raise flaps. This predisposes to ischaemia and frank flap necrosis occurs in some cases if vascular disruption has been excessive: The neuro-vascular bundle runs between the internal oblique muscle and the transversus, and enters the rectus sheath posterolaterally. Extensive unguarded dissection in this plane can easily damage these nerves and vessels. Surgical modifications to preserve vascular supply via periumbilical epigastric perforators have been described [31]. Skin necrosis must be treated urgently since it may ultimately expose the mesh and precipitate infection. Infection is a serious complication and is exacerbated by implantation of material, especially when foreign and non-biological. Infection must be treated aggressively because, when established, it usually culminates in mesh explantation [30]. Imaging is used to both detect and aspirate abscesses for microbiological diagnosis, and to guide drainage procedures. The main proposed advantage of biological mesh is that it is less prone to infection and can be used in fields that contained a previously infected non-biological prosthetic [32].

Later complications that are not specific to CVH repair include adhesions, bowel obstruction, abscess and enterocutaneous fistula: Their imaging and radiological management is no different than usual practice, accepting that this particular group of patients may pose difficulties due to their body habitus. As mentioned previously, intraperitoneal mesh is technically easy to place but is in direct contact with abdominal viscera, usually small bowel, and is therefore associated with small bowel adhesions, perforation and fistula.

Inevitably, the most common late recurrence is hernia recurrence. CVH involves difficult surgery, performed on difficult patients, and recurrence is unfortunately common, reaching approximately 30%, even in experienced hands. Visualisation of the mesh itself is often difficult on imaging because it is a thin material, closely opposed to adjacent structures. Detection is especially difficult if the mesh is a type that becomes incorporated postoperatively. While on imaging it is occasionally possible to identify mesh that has become separated at its periphery (Fig. 3), a laterally located recurrence will provide the clinical clue that hernia recurrence is due to peripheral detachment. Detachment is easiest to detect in those meshes that have been stapled or tacked at their periphery, since these fixators are identified easily on CT.

## Summary

Patients with CVH are referred increasingly for cross-sectional imaging and this review has attempted to familiarise radiologists with the pertinent surgical questions and the methods and procedures that are used in these patients. The hope is that radiologists will be better able to provide an informed report in this group of patients. Presently, imaging is most often deployed to determine preoperative hernia morphology and to identify and treat complications. However, it is likely that in the near future imaging will be requested to provide prognostic information regarding the type and extent of surgery needed to treat the hernia, and to predict the risk of its recurrence.

## Abbreviations

CVH: Complex ventral hernia
